# Careful with understudied phyla: The case of chaetognath

**DOI:** 10.1186/1471-2148-8-251

**Published:** 2008-09-17

**Authors:** Ferdinand Marlétaz, Yannick Le Parco

**Affiliations:** 1 Station Marine d'Endoume, CNRS UMR 6540 DIMAR, Centre d'Océanologie de Marseille, Université de la Méditerranée, Marseille, France

## Abstract

**Background:**

A recent study by Barthélémy *et al*. described a set of ribosomal protein (RP) genes extracted from a collection of expressed sequence tags (ESTs) of the chaetognath (arrow worm) *Spadella cephaloptera*. Three main conclusions were drawn in this paper. First, the authors stated that RP genes present paralogous copies, which have arisen through allopolyploidization. Second, they reported two alternate nucleotide stretches conserved within the 5' untranslated regions (UTR) of multiple ribosomal cDNAs and they suggested that these motifs are involved in the differential transcriptional regulation of paralogous RP genes. Third, they claimed that the phylogenetic position of chaetognaths could not be accurately inferred from a RP dataset because of the persistence of two problems: a long branch attraction (LBA) artefact and a compositional bias.

**Results:**

We reconsider here the results described in Barthélémy *et al*. and question the evidence on which they are based. We find that their evidence for paralogous copies relies on faulty PCR experiments since they attempted to amplify DNA fragments absent from the genomic template. Our PCR experiments proved that the conserved motifs in 5'UTRs that they targeted in their amplifications are added post-transcriptionally by a trans-splicing mechanism. Then, we showed that the lack of phylogenetic resolution observed by these authors is due to limited taxon sampling and not to LBA or to compositional bias. A ribosomal protein dataset thus fully supports the position of chaetognaths as sister group of all other protostomes. This reinterpretation demonstrates that the statements of Barthélémy *et al*. should be taken with caution because they rely on inaccurate evidence.

**Conclusion:**

The genomic study of an unconventional model organism is a meaningful approach to understand the evolution of animals. However, the previous study came to incorrect conclusions on the basis of experiments that omitted validation procedures.

## Background

Recently, Barthélémy *et al*. studied the ribosomal protein (RP) set of *Spadella cephaloptera *in order to investigate the phylogenetic and genomic features of chaetognaths [1]. Chaetognaths are small marine predators of significant importance in the planktonic ecosystem [2]. However, they are mainly known for their singular morphological and developmental characters that have been extensively debated by zoologists since the discovery of the phylum [3-6]. Furthermore, the molecular phylogeny of these organisms has also proved problematic, which makes their placement one of the most difficult issues in animal systematics [7,8]. Noticeably, two phylogenomic studies recently argued for the inclusion of chaetognaths in protostomes but proposed different branchings for this phylum: as the sister group of all other protostomes or as the sister group of just the lophotrochozoans [9,10]. The data analyzed by Barthélémy *et al*. [1] came from a collection of 10,000 ESTs that were sequenced by our team in order to perform phylogenomic inference, as described previously [9]. From this data, Barthélémy *et al*. retrieved the ESTs encoding the 78 ribosomal proteins, which are major components of the ribosome translation machinery [11]. These genes are broadly conserved during evolution and they have rarely been duplicated among eukaryotes [12]. Their analysis of this dataset of ribosomal protein transcripts lead Barthélémy *et al*. to emphasized three main findings [1].

• First, they identified nucleotide variants of certain RP genes in the surveyed ESTs. They stated that these variants correspond to duplicated, paralogous copies on the basis of PCR amplification of the alternative forms in single individuals. Accordingly, the authors proposed an allopolyploid origin for the chaetognath phylum. Moreover, they suggested that the two paralogous gene sets were differentially regulated depending on tissues and life-stages of *S. cephaloptera*.

• Then, they described conserved 28-nucleotide stretches at the extremity of 5'UTRs of unrelated RP transcripts. These motifs are overall similar but could be distinguished by some diagnostic residues, from which these forms were called TAC and TTT. They assumed that these motifs belong to the genomic copies of all these genes. They claimed to have identified binding sites for transcription factors in these motifs and thus proposed that these motifs are involved in the regulation of transcription.

• Finally, they performed phylogenetic inference from a concatenated RP dataset of ~5000 amino acid positions and 7 taxa, but were unable to accurately position chaetognaths among metazoans. The authors said that this lack of resolution could be due both to long-branch-attraction and to nucleotide compositional bias affecting the divergent chaetognath sequences.

Here, we prove that mistakes were introduced in this previous study, making these three conclusions incorrect. We first challenge the hypothesis that the conserved motifs they observed in 5'UTRs are present within the genomic copies of numerous unrelated RP genes. In contrast, we actually show that these motifs were added post-transcriptionally by a trans-splicing mechanism. Then, we investigate the possible causes for the lack of resolution of the phylogeny previously obtained. We point out that instead of the two biases mentioned by Barthélémy *et al*. [1], the problems encountered are incident to the limited set of taxa sampled.

## Results and discussion

### Experimental questioning of the gene duplication hypothesis

The exploration of transcript diversity within the EST collection revealed the presence of distinct variants of numerous ribosomal proteins, which are primarily distinguished by strong nucleotide divergences in both coding sequence and non-coding portions of the transcripts. At the extremity of the 5'UTRs of all these unrelated transcripts, Barthélémy *et al*. also detected two 28-nucleotides long motifs, which are overall similar but with the exception of some diagnostic residues that prompt them to call these motifs the TAC and TTT forms [1].

**Figure 1:**
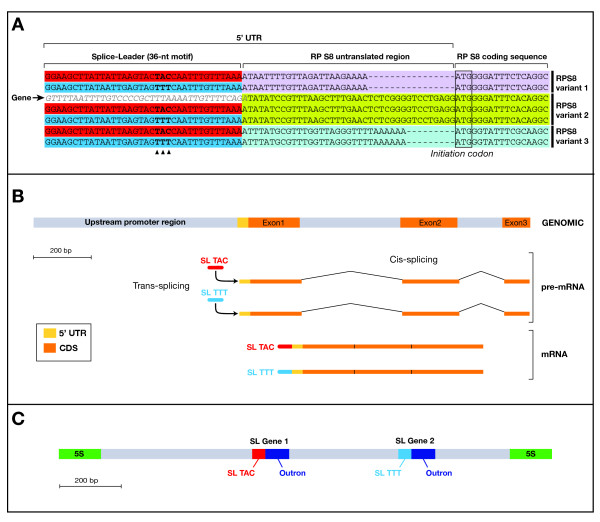
**Structure of ribosomal protein S8 gene and evidence for trans-splicing. **(A) Alignment of selected ESTs from three distinct variants of RP S8 with the genomic sequences of the Variant 2 of RP S8 (arrow, "gene"). Transcripts from each variant clearly show alternatively the TAC (red) and TTT (blue) motifs, which are absent from the corresponding genomic region. (B) The characterization of several PCR products and their comparison with EST sequences allowed us to determine the positions and lengths of the introns as well as those of the 5' UTR, the coding sequence (CDS) and the upstream region. The post-transcriptional addition of splice-leaders (SL) is schematized: the two alternative classes of SLs (TAC and TTT) have been retrieved in the RP S8 transcripts present in the library. Alignment corresponding to this schematic representation is available as Additional file 6. (C) The splice-leader genes encoding the two forms of splice-leaders are located in the 5S cluster region. Each splice-leader gene includes the splice-leader sequence and the outron, which is excised during the trans-splicing processing. Alignment corresponding to this schematic representation is available as Additional file 7.

Barthélémy *et al*. tested whether these ribosomal protein variants correspond to genes duplicated within the genome of *S. cephaloptera *by attempting to amplify these alternative variants from the genomic DNA of single individuals. For this purpose, they designed reverse primers in the coding sequence of the targeted genes and forward primers within the conserved 5'UTRs motifs that could be split in TAC and TTT forms, based upon divergent nucleotides (Figure 1A, TAC colored in red, TTT in blue). This experimental design relies on the hypothesis that these motifs are present in the genomic copies of the RP genes, in the same position as it lies in their transcripts. In their study, amplifications were performed for three different individuals and four genes (RP S8, S25, L15 and L27) that each included at least two variants (for example, RP S8 had three variants). It was claimed that some products were recovered, but no documentation was provided, neither sequences nor photos of the gel-electrophoresis results [1]. Nevertheless, the authors concluded that duplicated paralogous genes of ribosomal proteins are present in the genome of *S. cephaloptera*.

We argue here that the 5'UTR motifs in which Barthélémy *et al*. have selected their forward primers, are not located in the genomic regions of the targeted genes, but are only added after transcription during a trans-splicing maturation of the pre-mRNAs. Indeed, we noticed that these conserved motifs are present at the 5' end of ~30% of the transcripts in the library and that those transcripts code for many kinds of proteins, not just RPs (Additional file 1). We also found that, with a length of 36-nucleotides, these conserved motifs are slightly longer than the 28-nucleotides previously described, as illustrated by the example of RP S8 (Figure 1A). We consider that the occurrence of such conserved motifs in hundreds of distinct genes is inconceivable and we propose instead that this pattern reveals the occurrence of trans-splicing. The observation of similar stretches of conserved nucleotides at the 5' ends of transcripts from unrelated genes has been considered as firm evidence for trans-splicing in an abundant literature dealing with organisms from all over the tree of eukaryotes, such as euglenozoans, hydrozoans, nematodes, urochordates and rotifers [13-16]. The trans-splicing mechanism acts through spliceosomal addition at the 5' end of transcripts of a splice-leader (SL) sequence that is encoded by a distinct locus, the splice-leader gene. This splice-leader sequence is then found at the 5' end of a large subset of transcripts, but is found to be absent from the genes themselves that are related to these transcripts. Adding the splice-leader is said to ensure a better stability of trans-spliced transcripts, but trans-splicing is also associated with operonic transcription in some metazoans (e.g. urochordates and nematodes, see ref. [17]).

The occurrence of trans-splicing in *S. cephaloptera *was verified by our experimental attempts to reproduce and complete the work of Barthélémy *et al*. [1]. We first targeted the Variant 2 of the ribosomal protein S8 (RP S8) gene with the same set of oligonucleotide primers used in their study (Additional file 1 in [1]), but we were not able to amplify any product using the conditions given in the above publication (see methods). Conversely, using an alternate forward primer located in the coding sequence of the Variant 2 of RP S8 gene, we recovered the genomic sequence of Variant 2 of the RP S8 with the same DNA sample, which excluded any PCR problem in our attempt to reproduce the results of Barthélémy *et al*. (Figure 1B and Additional file 2). Further sequencing of this product allowed us to characterize the structure of RP S8 with the arrangement of its introns and exons (Figure 1B). Second, the absence of TAC and TTT motifs in the genomic sequence of the RP S8 gene was definitely proven by cloning and sequencing its upstream region using an original PCR genome walking strategy, that is, no such motifs were found here (Figure 1B). These two observations are consistent with the post-transcriptional addition of the TAC and TTT motifs by a trans-splicing mechanism. Finally, definitive evidence of trans-splicing was provided through the localization and characterization of the genes that encode splice-leaders corresponding to TAC and TTT motifs. We specifically surveyed the 5S cluster region using forward and reverse 5S primers, because this is the region that usually contains splice-leader genes in other species that perform trans-splicing [16]. In this way, we identified two SL genes organized in tandem within a unit of the 5S gene cluster (Figure 1C). These tandem genes correspond to the two SL motifs (TAC and TTT) and both include an outron, a cotranscribed sequence that is excised during trans-splicing process [18].

In summary, three lines of evidence strongly support the occurrence of trans-splicing in *S. cephaloptera*: first, the conservation of nucleotide stretches, the splice-leaders, at the 5'-end of unrelated transcripts; second, the lack of these splice-leader motifs in the genomic copies of considered genes; and third, the discovery of SL-genes within 5S clusters, where they usually lie in other species exhibiting trans-splicing.

We have demonstrated that TAC and TTT motifs could not be reliably used to demonstrate the presence of paralogs but the question remains of whether the variants of ribosomal proteins, such as the variants 1–3 of RP S8, correspond to paralogous genes or not (see [19]). Conceivably, such paralogs could have arisen either through previous whole-genome duplications or allopolyploidization events in the lineage leading to *S. cephaloptera*. Alternatively, they may simply not be paralogs but instead may result from cryptic-speciation. According to this latter view, the multiple arrow worms collected for library construction would belong to several different cryptic species within the sampled population. The nucleotide divergence observed between the variants of RP genes would then correspond to the genetic differentiation between these cryptic species. It has recently been discovered that cases of cryptic speciation are more common than originally expected [20] and cryptic speciation could actually be widespread among marine organisms, as illustrated by the example of the well-known *Ciona intestinalis *tunicate [21].

### Ribosomal proteins fully resolve chaetognath and animal phylogeny

Using a dataset of about 5000 amino acids and 7 taxa, Barthélémy *et al*. did not recover a clear positioning of the chaetognath taxon in the phylogenetic tree, despite the use of improved inference methods, in particular the site-heterogenous CAT model that generally lessens the problem of long-branch attraction [22]. Accordingly, they attributed this lack of resolution to some putative problems, namely, a persistent long-branch-attraction (LBA) artefact, a bias of amino-acid or nucleotide composition, a divergence between the variants of RP genes and finally the sampling of a too limited set of taxa. However, no attempt was made to test which of these many problems were the main culprits.

**Figure 2:**
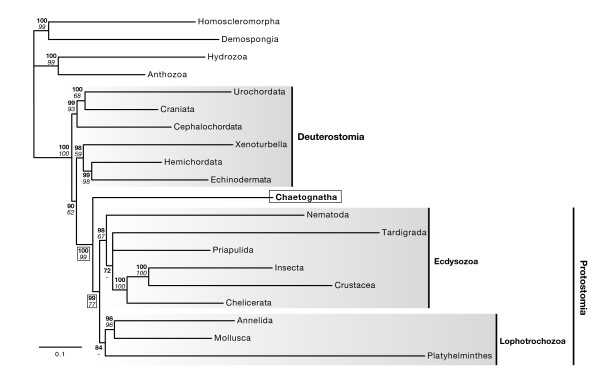
**Ribosomal protein dataset fully resolves animal phylogeny and reveals chaetognaths to be the sister taxon of all other protostomes.** This tree was inferred using phylobayes with the CAT model from a dataset of 12,764 amino acid positions after removal of ambiguous positions by Gblock. Bayesian posterior probabilities (upper numbers, bold type) and ML bootstrap support (lower numbers, italic type) are indicated as support values and circled for nodes relevant to chaetognath branching. Bayesian posterior probabilities over 95% and ML-bootstrap values over 70% are considered valid support.

Hence, we carried out several analyses that revealed that the main culprit was the scarce taxon sampling. First, we illustrated the power of a ribosomal protein dataset for resolving the phylogeny of bilaterians by employing the site-heterogenous CAT model on a dataset containing 12,764 aminoacid positions and 20 taxa (Additional file 3). We recovered the major clades of the so-called 'new animal phylogeny' with reliable support values [23,24] (Deuterostomia, Lophotrochozoa and Ecdysozoa, Figure 2) and we positioned the chaetognaths as the sister group of all other protostomes with significant posterior probabilities (PP 99%) and bootstrap support (BS 77%). However, the CAT-based analysis failed to recover the monophyly of the deuterostomes and instead united the hemichordates, echinoderms and xenoturbellids with the protostomes, but with low support (PP 90%) (Figure 2). This topology has been obtained by an independent study that used similar conditions [25], but it is clearly contradicted by morphological analyses and molecular analyses using site-homogenous model such as WAG, that is those analyses upheld deuterostome monophyly [26].

Systematic errors related to model violation, as well as problems due to limited taxon sampling, are well-known pitfalls in phylogenetic reconstructions, and numerous approaches allow the detection and resolution of these problems (e.g. see [27,28]). First, using a relative-rate test we excluded that chaetognaths evolved at a significantly higher rate than other taxa (p-value 0.087), which is, for instance, the case for platyhelminthes which did evolve faster (p-value 0.014). Hence, this testing supports our phylogenetic findings by excluding the possibility that our placement of chætognaths is an LBA artefact. Adding to this support, the CAT model we used in tree reconstruction itself minimizes LBA [22].

**Figure 3:**
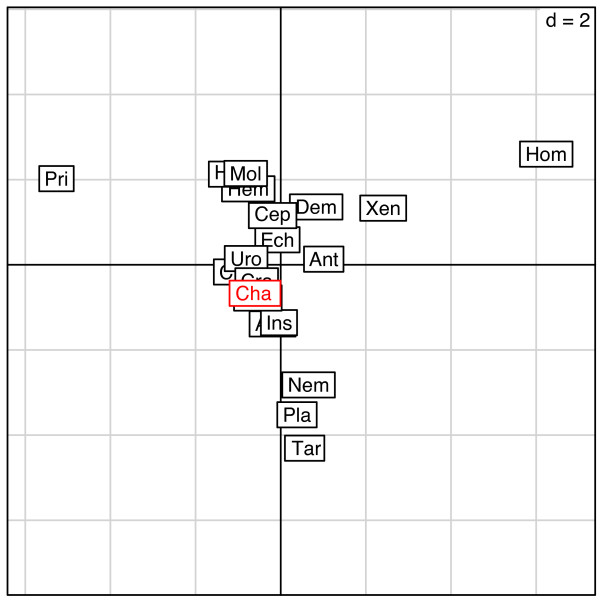
**Principal component analysis (PCA) of amino acid frequencies on the ribosomal protein dataset.** The first three letters of each taxon name are plotted onto the principal axis. Note the position of the chaetognaths (Cha), in red. The most divergent sequences, furthest from the center, are those of priapulids, homoscleromorphs, tardigrades, platyhelminthes and nematodes. Homoscleromorpha is the only taxon to fail the chi-square test of divergence from overall composition (Additional file 3).

Also, Barthélémy *et al*. suggested that a compositional bias impacts the phylogenetic placement of chætognaths. In dataset with such biases, taxa with high content in certain nucleotides or amino-acids (e.g. with G-C rich genes) can group together artefactually as they have independently evolved these biases through homoplasy. Compositional biases mainly affect the nucleotide datasets, but nucleotide composition could influence amino-acid content, so amino-acid datasets can have this bias too [29]. Therefore, we performed a principal component analysis and a test of amino acid composition to evaluate potential deviation in amino acid composition (Figure 3 and Additional file 3). We found that the amino acid composition of some taxa is strongly divergent (e.g., Priapulida, Homoscleromorpha) but that this is not the case for chaetognaths, whose content is utterly typical (p-value 0.906). Finally, we estimated the impact on the overall topology of the divergence between the variants of each ribosomal protein, which corresponds either to paralogous copies or to haplotypes from distinct cryptic species. For this purpose, we constructed alternate chaetognath taxa including either the most- or the least-divergent sequence variant of *S. cephaloptera *RP when these two forms are present (see Additional file 4). Trees inferred from alignments including any of these taxa resulted in identical topologies and similar support values (Additional file 5), which excludes the possibility that our previous statements were biased by the inclusion of one of the distinct gene variants [9].

In summary, we excluded three of the potential pitfalls that could misplace chaetognaths in the RP-based phylogenetic tree: a remnant long-branch attraction effect, a compositional bias of amino-acids, and an intra-taxon molecular divergence. Unaddressed, these problems could have raised doubts about our present finding as well as those of previous studies [9,10]. Our analyses clearly argue for the chaetognaths as the sister group of all other protostomes, as is supported by both site-heterogenous (CAT) and classical site-homogenous (WAG) models [9]. Interestingly, this phylogenetic position was independently recovered in two recent studies, mainly based on ribosomal proteins that include enlarged taxonomic sampling with acoel flatworms [30] or bryozoans and syndermates [31].

Here, the position of chaetognaths was primarily resolved by improving the taxon sampling, from 7 to 20 major taxa. The problem of inadequate taxon sampling has been at the the root of major controversies in the phylogenomic field [24,31,32]. Studies based on a limited taxon sampling have proposed the revival of the coelomata hypothesis [33,34] or even proposed the impossibility of resolving animal phylogeny as a whole [35], but careful examination of these results later revealed that the authors had been misled by incomplete taxon sampling [24,32].

## Conclusion

We show here that the study by Barthélémy *et al*. [1] relies on a set of evidence whose accuracy is dubious. Particularly, the major claim, for extensive gene duplication, was made on the basis of experiments and analyses that did not include sufficient validation procedures. For example, no sequences or gel photographs were provided to support the results of PCR amplifications of genes. Hence, we suggest that careful precautions be taken when dealing with understudied model organisms for which only limited data and few preliminary studies are available. The originality and the novelty of chaetognath genomics deserve data and analyses of the highest grade. Further investigation of the questions briefly evoked in the present paper will be the subject of a new publication designed to enlighten the genomic features of the astonishing organisms that are chætognaths [19].

## Methods

### PCR amplifications

Genomic DNA template was isolated using the Wizard SV Kit (Promega) from *Spadella cephaloptera *adult specimens collected in the Calanque of Sormiou near Marseille, France. The melting temperatures were determined (MacVector software) for each pair of primers as indicated in Additional file 6. Melting temperature (Tm) was set at 50°C for targeting 5S cluster units (primers 5SFor and Rev) and at 59°C for amplifications of RP S8 (primers TAC, TTT, S82R and S8-2F2). Cycling conditions were as follows: 94°C for 1 minute, Tm for 1 min and 72°C for 2 min. The PCR products were subsequently checked using gel electrophoresis, cloned in pGEM-T easy (Promega) and sequenced by Genome Express (Grenoble, France).

### Genome Walking procedure

PCR-based genome walking was carried out as described in [36] using a set of semi-degenerate forward primers (named semi in Additional file 6) and specific nested reverse primers matching the first exon of RP S8 (Additional file 2). Three rounds of amplifications were conducted with the four specific nested primers using the touch-down strategy (Tm ranging from 60°C to 41°C). Several independent products were cloned and sequenced (Additional file 2).

### Phylogenomic-class dataset assembly

The ribosomal protein dataset was constructed using the composite database strategy, as described in [9]. Available sequences from a wide range of animal taxa including newly published sequences were extracted from the NCBI EST database [37] using the BLAST program [38]. Sequences were compiled for 20 taxa, mostly as chimeras, as detailed in Additional file 3. Data parsing, conceptual translation of ESTs and assembly of the concatenated alignments of amino acid positions were performed using computer scripts based on the Bioperl framework [39]. The alignment was manually refined using MacVector (Accelrys) and ambiguously aligned regions were then removed using GBlocks [40]. This processing yielded an alignment with 12,764 amino acid positions and 20 taxa that is available upon request yannick.leparco@univmed.fr.

### Phylogenetic inference

The ribosomal protein dataset was analyzed using Phylobayes 2.3 implementing the CAT model, which accounts for site-heterogenous amino acid substitution processes [41]. Burn-in period was determined by plotting parameters across all runs and stationary state was checked by comparing the frequencies of bipartition between several independent runs [22]. The maximum-likelihood tree was inferred using Treefinder [42] assuming a WAG+Γ_4_+F model and support was estimated through 100 non-parametric bootstrap replicates.

### Evaluation of potential biases

In order to evaluate the impact of variants of RP genes detected in *S. cephaloptera*, two alternate datasets including either the most or the least divergent variant of ribosomal proteins from *S. cephaloptera*, were assembled. Independent alignments were built that include the sequences of the variants of RP genes from *S. cephaloptera *and when available the sequences of another chaetognath species, *Flaccisagitta enflata *(Additional file 4). For each of these alignments, phylogenetic trees were inferred (PhyML, WAG+I+Γ4 [43]) and patristic distances were computed for each of the different chaetognath sequences (Additional file 4). These values allowed us to select the most and the least diverging ones.

Principal component analysis was carried out to evaluate the extent of amino acid compositional heterogeneity using the R statistical package and ade4 library. Chi-square tests of deviation from average amino acid content in the dataset were also computed using Tree-puzzle [44].

The relative rate test assessing differences among the evolutionary rates of selected lineages, was performed using RRTree [45].

## Authors' contributions

Both FM and YLP participated in the conception, design, analysis, interpretation of data, and drafting of the manuscript. Both authors read and approved the final manuscript.

## Response 1

Jean-Paul Casanova, Roxane M Barthélémy, Eric Faure§

LATP, UMR 6632, Evolution biologique et modélisation, Case 5, 3 Place V. Hugo, Université de Provence, 13331 Marseille cedex 3, France

^§^Corresponding author

Email addresses:

EF: Eric.Faure@univ-provence.fr

JPC: bioplank@univ-provence.fr

RMB: Roxane.Barthelemy@univ-provence.fr

### Introduction

This debate cannot be understood out of its context. One of us (JPC), in his description of the new genus *Archeterokrohnia *Casanova, 1986 [46], hypothesized possible relationships with molluscs and wrote (p. 193, English translation): "*the main difficulty [of this hypothesis] seems to lie in the fact that chaetognaths have a deuterostomian type of development while molluscs are protostomian*" (chaetognaths were then said to be deuterostomes). So, he pointed out the interest of the chaetognath model [47] and submitted a project of sequencing of their genome, in collaboration with Dr P. Pontarotti (Marseilles, France), that was accepted (2000) by the Génoscope-Centre National de Séquençage, and two posters were rapidly produced [48,49]. Then, two members of his lab joined Le Parco's team; that explains why now two teams in Marseilles work on chaetognaths which status of "*protostome with deuterostome-like development*" has been evidenced at the molecular level by Le Parco's team [9], confirming thus unambiguously previous Casanova's hypothesis of the protostomian nature of the phylum based on morphological and anatomical data. So, one cannot be astonished to read two papers on the mitochondrial genome of *Spadella cephaloptera *Busch, 1851 [50,51], and two others on ribosomal protein sequences issued from the same ESTs data set [9,1]. Our findings of several interesting features such as the presence of ribosomal protein (RP) paralogous sequences and of specific sequences in 5' UTR regions [1], that were not reported in the publication of Le Parco's team [9], is the matter of this debate.

Our response is divided into two parts, the one concerning molecular phylogenetic analyses by Dr A. Chenuil is in a separate report.

### Scientific critics addressed to our paper

We shall consider here all the scientific critics other than the phylogenetic ones addressed to our paper that Marletaz and Le Parco wholly contest in their manuscript above. When they wrote *"Indeed, we noticed that these conserved motifs are present at the 5' end of ~30% of the transcripts in the library and that those transcripts do not only derive from RP genes"*, we simply reply that this feature was not mentioned in their publication of 2006 [9]. Moreover, they have distorted the thought of our article, for example, when they say in the Background "*They *[us]*claimed they have identified binding sites for transcription factors in these motifs and thus proposed that these motifs are involved in the regulation of transcription*", whereas we have only suggested this, writing "*two novel and highly conserved elements have been identified (5'-TAATTGAGTAGTTT-3' and 5'-TATTAAGTACTAC-3') which could correspond to different transcription factor binding sites on paralog RP genes"*. This is based on the fact that we have used several prediction programs in order to search if these regions could correspond to "consensus" sequences previously known. Two programs have indicated that a part of these regions is similar to transcription factor binding sites. Moreover, in some genes, DNA binding site(s) have been identified in the downstream region of the transcriptional start site [52,53].

Marlétaz and le Parco note also in their manuscript above: *"We find that their evidence for paralogous copies relies on faulty PCR experiments since they attempted to amplify DNA fragments absent from the genomic template. Our PCR experiments proved that the conserved motifs in 5'UTRs that they targeted in their amplifications are added post-transcriptionally by a trans-splicing mechanism"*. A trans-splicing leader mechanism is a possible interesting hypothesis, but only supported by one example. Moreover, the authors have only made PCRs on one form of RPS8 gene that already gives one plenty to think about the rejection of our finding of paralogy and duplication of the genome. This agrees with the fact that they did not mention these events in their publication of 2006 [9] dealing with the same data set.

If all the 5'UTR regions are added on ribosomal protein mRNAs through a trans-splicing mechanism, how to explain our PCR results? There are three non-exclusive explanations if this hypothesis is true:

- Possible chimeric PCR products (5' part corresponding to the DNA regions containing the trans-splicing sequences and 3' part to a ribosomal protein gene ORF) could be amplified; however, it is relatively improbable to obtain sizes similar to those of corresponding processed mRNAs.

The two following explanations are more probable.

- Marletaz and Le Parco in their manuscript above have sampled a cryptic species or even a well different species for their experiments (see below). Such an explanation for their differences in PCR results cannot be totally ruled out when one knows the great variability of musculature, the main chaetognath body component, in relation with habitat and behavior [54].

- Our PCR results would be the amplifications of RP processed pseudogenes. It is widely recognized that in animals most if not all of the RP genes have a number of processed pseudogenes located elsewhere in the genome [55]. For example, in mammalian cells, a single gene encodes each RP but this gene generates a large number (10–20 copies) of silent, processed pseudogenes [56]. Moreover, generally, the number of pseudogenes is greater in multigenic families. Amplifications of processed pseudogenes can generate amplicons with the expected size versus the homologous EST sequences. So, sequences of the amplicons would have not given interesting data since they would have been similar to those of the mRNA sequences; only careful analyses of important genomic data such as a BAC collection could allow responding unambiguously to this question and we had not these data. Moreover, in their *S. cephaloptera *EST collection, Marletaz et al. [9] have found that some sequences are similar to those of reverse transcriptase genes or of parts of retrotransposons; similarly, LINE-like and *Gypsy*-like retrotransposons have been found in *Sagitta sp*. [57] and in *S. cephaloptera *[58], showing evidence of the presence of functional reverse transcriptase proteins in chaetognath cells. Interestingly, SINE elements are retrosequences; this group contains all the sequences arisen by reverse transcription of ribosomal, messenger and small stable RNAs [59,60]. These elements are non-autonomous for transposition, and as they do not contain reverse transcriptase gene, they rely on the activity of reverse transcriptase proteins encoded by LINEs to retrotranspose [61]. This mechanism can explain the origin of numerous pseudogenes. Lastly, as we have used reverse primers specific of each type of paralogous genes, positive PCRs in the three hypotheses exposed above evidence the presence of these paralogs.

### Remarks on Marlétaz and Le Parco's work

#### Biological material

Why have we made PCR experiments? Because inter-individual or even inter-specific variations if cryptic species are involved (see below) could not be excluded since the EST library has been constructed with several individuals originating from two distinct separated populations, in the Brusc Lagoon and the open sea, off La Ciotat. This is rather unusual in genomics. In view to question the existence of paralogous genes arisen through genome duplication, Marlétaz and le Parco in their manuscript above wrote: *"However, alternative hypotheses such as the cryptic-speciation hypothesis cannot be ruled out. This latter hypothesis states that the multiple arrow worms collected for library construction would belong to several cryptic species spread within the sampled population. The nucleotide divergence observed between the variants of RP genes would then correspond to the genetic differentiation between these cryptic species. It has recently been discovered that cases of cryptic speciation are more abundant than originally expected *[19]*and cryptic speciation could actually be widespread among marine organisms"*. It would have been more appropriate to cite previous works dealing with this problem by one of us (JPC) [62,63]. Indeed, in the coastal area of Marseilles, a few *Spadella *specimens have been described which yet remain to be assigned to two distinct species, notably in the numerous submarine caves out of which they can sometimes be found among *S. cephaloptera*. It can be recalled here that some others resembling *Spadella ledoyeri *Casanova, 1986 were later raised to the specific rank [64]. Thus, it would have been more judicious to make the EST collection using a single individual of the species *Sagitta lyra *Krohn, 1853 (size > 40 mm versus 3–4 mm for *S. cephaloptera*) commonly found off Marseilles, in order to avoid this type of problem!

Moreover, Marlétaz and Le Parco used specimens (number unknown) from a third origin (Calanque of Sormiou) for their present PCR experiments, an area known for its numerous submarine caves. Until then, one of us (JPC) was asked to help their identification, e.g., in 2006 he was yet acknowledged by Le Parco [7] "*for providing some specimens and for helping *[them]*in species identification and scientific comments"*. Thus, we are not sure of the real nature of their specimens: *Spadella cephaloptera? *Specimens of a cave *Spadella *or even of *S. birostrata *Casanova, 1987, living at depths = -146 m off the French Mediterranean coasts [65], but which might be able to reach neritic waters during the cold season when the temperatures of the water column are homogenized, as commonly observed for numerous deep organisms? Moreover, highly divergent mitochondrial lineages have been found in sympatric Mediterranean populations of the planktonic chaetognath *Sagitta setosa *Müller, 1847, suggesting to Peijnenburg et al. [66] the existence of unidentified sympatric sibling species. That is why we have always sampled specimens in the Brusc Lagoon, either for our previous 18S and 28S rRNA experiments [67-69] or RP genes PCR [1]. Indeed, this population of *S. cephaloptera *is regularly studied by us since 1996 [70] and seems to be relatively isolated from populations of the open sea. The necessity to work on this isolated lagoon population has been an evidence for us since Riggio and Chemello's synthesis [71] on the role of coastal lagoons in genetic isolation and evolution of numerous of benthic taxa, based on the observation in a Sicilian lagoon of a higher ratio of endermism as compared to the whole Mediterranean Basin.

#### General remark on Marlétaz and Le Parco's phylogeny

Marlétaz and Le Parco in their manuscript above state: "*ribosomal proteins fully resolve chaetognath and animal phylogeny*". They must be careful with such an assertion and remember the different positionings they found according to the criteria they used when they wrote, for example, in 2004 [50]:*"It should be noticed that the inclusion of Chaetognatha within protostomes, based on mitochondrial data, is conflicting with *[their]*previous conclusions, based on Hox genes" *[72]).

#### Submission in databases

These authors should submit their EST collection as it is the use. On April 2006, ESTs sequences were under the accession numbers (AC) *CR940385 to CR954140 *on the EMBL database. On November 2007, 1001 of these sequences were again submitted under other numbers. For example, now, there are two ACs for the EST sequence corresponding to the cDNA clone 28YH18 (RPS8): CR952852 and CU563934. Moreover, in Table S4, the authors give a list of ribosomal proteins available in the chaetognaths *Flaccisagitta enflata *Grassi, 1881 and *S. cephaloptera*, but the amino acid sequences are not provided. This is rather embarrassing for readers and rather curious. Moreover, curiously again, although the authors wrote *"This processing yielded an alignment with 12,764 amino acid positions and 20 taxa that is available upon request yannick.leparco@univmed.fr)"*, they have refused to send it to us in spite of our request.

#### Comment on Figure 1 in the manuscript above

Marlétaz and Le Parco do not give the nuclear 3' terminal region of the RPS8 type II gene which could reveal interesting features; for example, some of our unpublished studies have shown that intergenic regions are sometimes very short, evoking a very probable operon structure. In eucaryotes, operons appear to be relatively less frequent than in procaryotes; however, some examples are well known [73,74]. We suggest to the authors to look for them in their data; it would be a pity to miss them (if any).

#### Careful with the understudied phylum Chaetognatha

We totally agree with one of the authors' final remarks that *"careful precautions should be taken when dealing with understudied model organisms for which only limited data and few preliminary studies are available*". Perhaps, this advice cannot be addressed to our team, since one of us (JPC) has a background knowledge of the phylum: about 60 publications on the systematics and general biology of the phylum, description of about a quarter of the extant species, and reports of many original observations such as the progressive stages of acquisition of one pair of appendages on the posterior half of the tail by modifying a part of their balancing fins, or their highly diversified musculature [54,75]. Because of this knowledge, we were astonished to read that the mitochondrial genome of *Spadella cephaloptera *[50] lacked the tRNA^Met ^gene found in *Paraspadella gotoi *Casanova, 1990 [76]. As these two genera are very related (they were only separated in 1986), we logically thought this gene was perhaps missed by Le Parco's team. This was right and we compared these two genomes [51] to go farther studying this model phylum. Our working methods are different, sometimes conflicting, but considered together they lead to a better knowledge of this interesting phylum. This is also true for the ribosomal protein analyses.

### Conclusion

Marlétaz and Le Parco have proposed an interesting alternative hypothesis concerning the 5'UTR region we discovered, but this needs supplementary data since supported by a sole example. For the same reason, the BAC sequences they have (started in 2001 in P. Pontarotti's lab) must be submitted to sequence database in order to verify the existence of numerous other examples of genes whose mRNA would be trans-spliced. A last question arises: why Marlétaz and Le Parco's critics are not exposed in their submitted manuscript? The proofs of the unreality of both paralogy of the ribosomal protein genes and polyploidy in chaetognaths they strongly suggest in this debate would then have been more convincing and, consequently indisputable. This remark is indirectly supported by the authors themselves when they conclude *"Further investigation of the questions briefly evoked in the present paper will be the subject of a new publication ... (Marlétaz et al. submitted)"*. Since this *"present paper" *is only a brief copy of the announced "new publication", it is therefore useless.

### Addendum

This debate began on November 2007 after the publication of our paper on August 27, 2007 [1]. It was useless. Indeed, the paper announced as 'submitted' by Marletaz and Le Parco (above) has been online since June 2, 2008 [77]. It confirms the existence of paralogy and polyploidy in *Spadella cephaloptera *that they missed in their previous paper [9] and which we discovered one year ago [1] studying the same EST collection. But our paper is not mentioned in their paper published in *Genome Biology *earlier this year [77]. Is this with a view to letting readers believe it is their discovery? Moreover, we suspected the existence of an operon structure when looking at their Fig. 1 in the manuscript above. They confirm this fact [77], but without an undisputable demonstration as we suggested (see above). No more comments are needed about such behaviour!

## Response 2

By Anne Chenuil

E-mail: anne.chenuil-maurel@univmed.fr

Address: Laboratoire DIMAR, CNRS UMR 6540. Université de la Méditerranée Aix-Marseille II, Centre d'Océanologie de Marseille, rue de la batterie des Lions, 13007 Marseille. France.

### Aspects related to molecular phylogenetic analyses and the long branch artefact

I first explain my implication in the debated publication: I was contacted by EF, one of the three authors (EF, RB and JPC) of a previous submitted version of the manuscript which I ignored, after one referee asked for more elaborate phylogenetic analyses, using nucleotide data and ML methods; I then asked Samuel Blanquart for his help for the Bayesian analyses he had recently developped and the CAT model.

The phylogenetic analyses in Barthelemy et al. (2007) [1] were neither a primary nor a major goal of the paper but we thoroughly explored elaborate methods attempting to avoid certain biases in reconstructing phylogenetic trees. We referred to the previous work of Marletaz et al. (2006) [9] several times and always in a positive way. Based on our results, we finally concluded that the lack of phylogenetic resolution in our data set was very likely to be due to limited taxon sampling associated with the long branch attraction artifact (LBA) and made the following prediction: "*... improvements to infer phylogenetic relationships of the chaetognath phylum will rely on using the PhyloBayes program with the CAT model on a wider taxonomic dataset than the one we used in the present study, such as that of Marletaz et al. 2006*". Marletaz and Le Parco (2008) in their manuscript above used the method we recommended and obtained a topology where chaetognaths branch at the base of protostomes with a high support though the support we obtained was low.

However, these authors wrongly summarize our phylogenetic analyses [1] and suggest that we ignored the problem of taxon sampling. They deplore that we considered the compositional bias as a potential artifact without assessing its importance, although we actually used dedicated probabilistic methods, and we concluded that *"...the LBA artifact seems to affect our phylogenetic reconstruction more than the base composition bias since the methods which are supposed to "correct" for GC-content variation among lineages do not change the topology obtained with more standard methods, while the method supposed to correct for LBA does change it*." [1]. Etc.

More importantly, I contest some aspects of their analyses in their manuscript above, in particular the distinctions made among factors causing lack of robustness, and their interpretation of a classical test.

a/Marletaz and Le Parco oppose the bias due to limited taxon sampling to other biases such as LBA, writing: "*we showed that the lack of phylogenetic resolution observed by these authors is due to limited taxon sampling and not to LBA or to compositional bias*" (see the manuscript above). This reasoning seems awkward, since, when long sequences are available as in the present case, limited taxon sampling is not a problem *per se *but only when there is systematic error due to non phylogenetic signal (such as LBA or base composition heterogeneity, or when the model of evolution does not correspond to the reality as e.g. when changes of model parameters occur within internal branches) impeding convergence to the true tree, even if an infinite number of characters is available. Furthermore, they used not only more taxa, but also many more amino-acid positions (12764) than we did (4638) [1], which makes their "demonstration" that taxon sampling is "the single problem" methodologically unconvincing. The increased support they obtained may result from increased sequence length as well as from improved taxon sampling ... associated with non phylogenetic signal.

b/They wrote that we did not assess the presence of biases properly, and that they proved that these biases do not apply. I contest these two points and will now explain why for the case of the LBA. They carried out a relative rate test (using the RRTree program) to compare the chaetognath sequence to the other ones (considered globally) and obtained a P-value of 0.087, stating that it excludes *"...the possibility that the current branching of chaetognaths is an LBA artefact*." First, the point is not to show whether or not chaetognaths evolve significantly more rapidly than the average of the other species, but to assess whether rate variation among lineages may generate spurious phylogenetic relationships. For the latter purpose, the chaetognath sequence should be compared with the other taxa individually, as we did [1], not to a group of species that includes taxa well known for their very high evolutionary rates (this is the case of, at least, the platyhelminth, as Marletaz and Le Parco themselves emphasized in their manuscript above). Second, the absence of proof (non significance) is not the proof of an absence. In addition, if, for the relative rate test, they had compared the chaetognath to a group of species after removing the very fastly evolving platyhelminth, it is likely that the P-value would have been much smaller than 0.087 (which is nevertheless nearly significant) as I show below. Although these authors seem to have overlooked this, we had performed Tajima's relative rate tests and reported that chaetognaths were much more rapidly evolving than molluscs "*suggesting that the union of the fast-evolving *Drosophila *and the fast-evolving chaetognaths may be an LBA artifact*" [1]. Once established that chaetognaths are not deuterostomes, and considering the sponge as the outgroup, in our 6 taxa data set, the question reduced to determining the rooted topology of three taxa: the chaetognath, the mollusc and *drosophila*. Deuterostomes were represented by one vertebrate and one echinoderm.

I now present the results of these relative rate tests with more details than in our initial study. Using the Mega software, we found a very significantly (P < 0.001) higher rate of evolution in the chaetognath relative to each sequence of our data set, except *Drosophila*, whatever the outgroup (sponge or yeast) and the type of substitution considered, either with the amino-acid or with the nucleotide data set. Compared to *Drosophila*, the higher number of substitutions in the chaetognath was either less significant (P-value between 0.03 and 0.048) or, in one case (nucleotide data, yeast outgroup), non significant. These results strongly support that spurious phylogenetic relationships tending to place chaetognaths at a basal position (and eventually to relate chaetognaths with arthropods) may occur artefactually from this dataset.

Citing one of Le Parco's paper [50] "*this long-branch attraction problem *[in chaetognath molecular phylogenetics]*is well documented and reviewed in the study by Mallatt and Winchell (2002)"*. In conclusion, I contest the assertion of Marletaz & Le Parco in their manuscript above that chaetognaths are not prone to long branch artefacts in the ribosomal protein data set.

## Supplementary Material

Additional file 7 **Unit of the 5S cluster including the two splice-leader genes organized in tandem, the splice-leader sequences and the outrons.** Primers employed for amplification are also specified. Click here for file

Additional file 1 **Examples of unrelated trans-spliced genes retrieved in the *S. cephaloptera *EST library.** The presence of the TAC and TTT classes of splice-leaders is indicated for both ribosomal protein genes and other genes. Click here for file

Additional file 2 **Structure of the ribosomal protein S8 gene with alignment of used primers, transcript sequences from the EST library and genomic sequence deduced from sequencing of PCR products.** The alignment especially stresses the absence of the splice-leader sequence from the 5'UTR and upstream promoter region. Click here for file

Additional file 3 **Description of the composite taxa employed in phylogenomic analysis.** The amount of missing data was determined for the final Gblocked alignment of 12,764 amino acid positions. p-values are from the chi-square test, which tests whether the amino acid composition of each sequence is identical to the average amino acid composition of the whole alignment. The number of species included in the taxons is specified, as well as the name of the principal species. Click here for file

Additional file 4 **List of ribosomal proteins available in the chaetognaths *Flaccisagitta enflata *(*Fe*) and *Spadella cephaloptera (Sc) *and their divergence rates.** The molecular divergence of each alternative form is indicated as ML patristic distance to the root (assuming WAG+Γ_4_+I) as well as the content of best contig (least divergient of all species and variants), shorter contig (least divergent variant of *S. cephaloptera*) and longer contig (most divergent variant of *S. cephaloptera*). Click here for file

Additional file 5 **Bayesian trees calculated using Phylobayes implementing the CAT model for the shorter contig (A), longer contig (B) and both of them (C) from *S. cephaloptera*, as described in Additional file 3.** Significant posterior probability values (pp > 0.95) are retrieved for all nodes and only lower ones are displayed in plain text. Both topology and support values remain unaffected by the use of variants of ribosomal proteins. Click here for file

Additional file 6 PCR Primers and annealing conditions used for PCR experiments. Click here for file
