# Supplementary figures and images for: Careful with understudied phyla: The case of chaetognath
Source: BMC Evol Biol. 2008 Sep 17;8:251. doi: 10.1186/1471-2148-8-251 (PMC2566580; doi:10.1186/1471-2148-8-251)

Figure S1

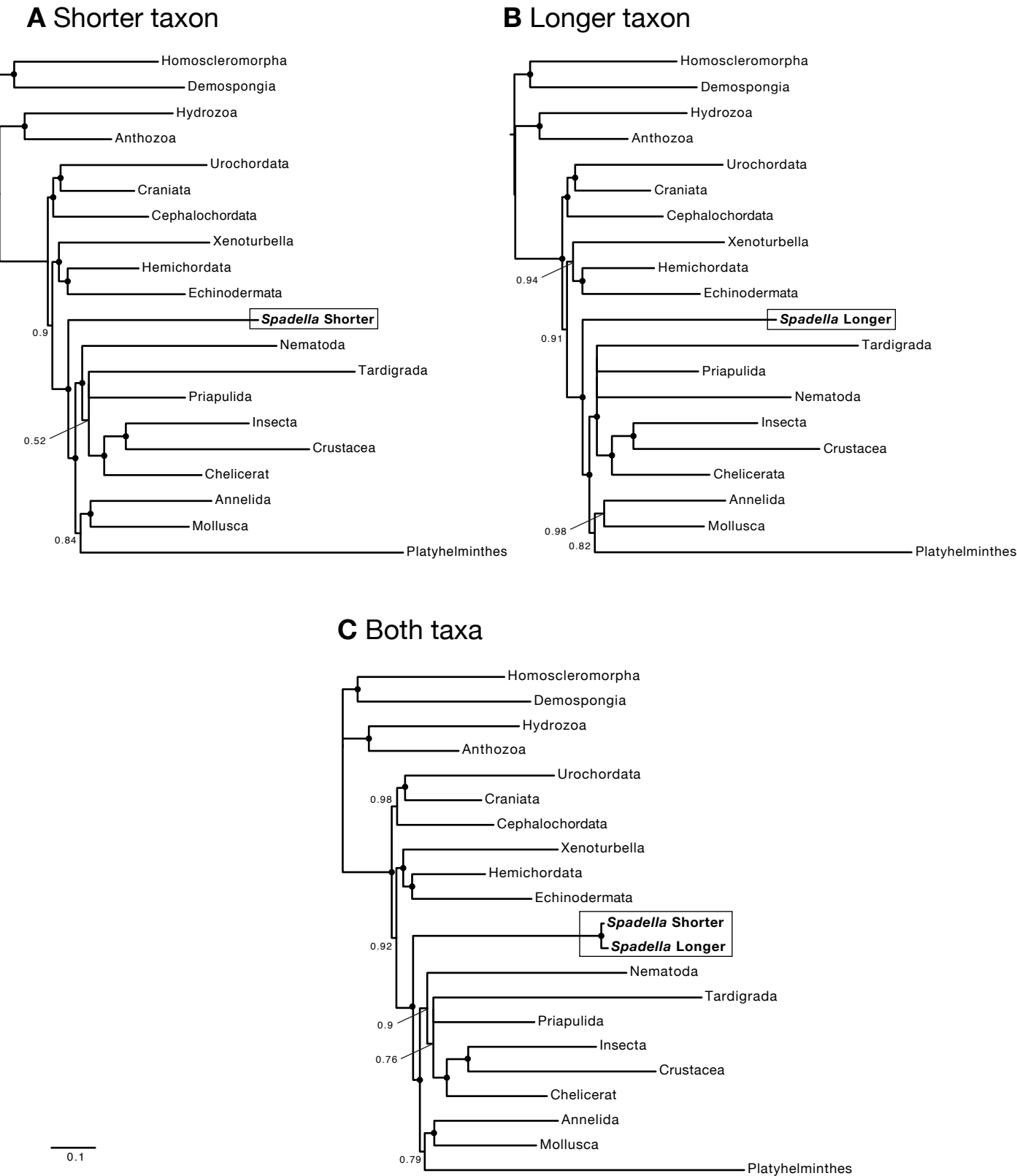

Supplement: Additional file 5 — Bayesian trees calculated using Phylobayes implementing the CAT model for the shorter contig (A), longer contig (B) and both of them (C) from S. cephaloptera, as described in Additional file 3. Significant posterior probability values (pp > 0.95) are retrieved for all nodes and only lower ones are displayed in plain text. Both topology and support values remain unaffected by the use of variants of ribosomal proteins. [file 1471-2148-8-251-S5.pdf]
